# A Comparative Analysis of Chemical, Thermal, and Mechanical Post-Process of Fused Filament Fabricated Polyetherimide Parts for Surface Quality Enhancement

**DOI:** 10.3390/ma14195880

**Published:** 2021-10-08

**Authors:** Ariadna Chueca de Bruijn, Giovanni Gómez-Gras, Marco A. Pérez

**Affiliations:** IQS School of Engineering, Universitat Ramon Llull, Via Augusta 390, 08017 Barcelona, Spain; ariadnachuecad@iqs.url.edu (A.C.d.B.); marcoantonio.perez@iqs.url.edu (M.A.P.)

**Keywords:** additive manufacturing, fused filament fabrication, PEI Ultem 9085, postprocessing, finishing operations, surface enhancement, vapor smoothing, thermal annealing, abrasive shot blasting, shot peening

## Abstract

Additive manufacturing technologies are increasingly being used in production systems because they shorten product development time and production cost, but surface integrity remains a limitation to meet the standards set by conventional manufacturing. In this research article, two chemical, one thermal, and three mechanical finishing operations are proposed to post-process fused filament fabricated Ultem 9085 parts. Their effects on the parts’ surface quality and dimensional accuracy (changes in their width, height, length, and mass) are examined through optical and electron scanning microscopy, and the advantages and disadvantages of each method are discussed. Microscope evaluation has proven to be a powerful tool to observe apparent differences and understand the nature of different morphological changes. Results indicate that chemical and thermal treatments and ball burnishing are good candidates to significantly enhance the finish of the parts, despite requiring the use of solvents or provoking dimensional changes to the parts. The effects of abrasive mechanical treatments are more moderate at a macroscopic scale, but the surface of the filaments suffers the most remarkable changes.

## 1. Introduction

The surface characteristics of a component determine how it will interact with its environment. In some cases, irregularities on the surface will constitute weak regions where cracks or corrosion may start to nucleate. Therefore, surface roughness could be a good indicator of the potential mechanical performance of a part [1]. In other cases, however, specific roughness values may be desirable to enhance the adhesion of cosmetic or functional finish coatings such as painting or metal plating [2].

Within the specific context of additive manufacturing (AM), the layer-by-layer material deposition that is characteristic of these technologies creates an uneven surface profile known as “stair-stepping effect” [3,4]. This issue poses a challenge in terms of superficial integrity and dimensional accuracy and has been recognized as a major concern in employing AM technologies for final part applications [5]. For this reason, monitoring, modeling, and compensation for surface roughness in AM have become popular fields of research [6,7,8,9,10,11,12].

The reviewed literature reveals that the most common approach to address this subject consists of optimizing pre-printing parameters, including the slicing strategy, raster angle, part orientation, infill percentage, printing temperature, and layer thickness. In this sense, Boschetto et al. [13] proposed a geometrical model of the filament that considers the radius and spacing of the profile section and can predict the dimensional deviations of acrylonitrile butadiene styrene (ABS) fused filament fabricated (FFF) parts as a function of the layer thickness and deposition angle. Their findings correlate with those published by Pérez et al. [14] and Buj-Corral [15]. The former performed an experimental study with polylactic acid (PLA) samples and found that layer height and wall thickness are the most important factors controlling surface roughness. The latter presented a geometrical model for the simulation of roughness profiles obtained with different print orientation angles in FFF PLA specimens and compared it to experimental results. Their findings were that roughness values increase with print orientation angle as the stair-stepping effect is accentuated.

Despite accurate optimization of process parameters, the desired surface quality of parts may not be achieved, or perhaps only a fraction of the surface needs to be conditioned to meet the end customer’s specifications. Thus, post-processing techniques constitute a complementary tool to refine the finish of additively manufactured parts [16]. In broad terms, these processes can be grouped into thermochemical and mechanical treatments. Thermochemical treatments take advantage of chemical substances or the application of general or localized heat to smooth the part’s surface. These methods include vapor smoothing, painting, electroplating or metallization, annealing, and laser finishing.

Many research works have investigated the vapor smoothing process; it is a relatively straightforward and well-established process. Chohan et al. [17,18,19] published a series of articles where they performed a parametric optimization to treat FFF ABS hip replicas with acetone vapors. They evaluated the impact of smoothing duration and repetition of smoothing cycles on surface finish, dimensional accuracy, and stability of the parts, and they concluded that small smoothing duration (30 s) and repeated cycles could yield remarkably lower surface roughness. They also developed a mathematical model for the prediction of the average surface roughness of the treated parts. Mu et al. [20] compared the effect of different mixtures of acetone and ethyl acetate to improve the surface coarseness of ABS specimens with different building orientations and concluded that the tensile strength of samples treated with the acetone or the mixed vapor decreased with increasing the exposure time. The best results in terms of mechanical performance were obtained when vapors of pure ethyl acetate were used. Jin et al. [21] and Rajan et al. [22] explored the use of tetrahydrofuran and dichloromethane, respectively, to smooth the surface and improve the toughness of PLA specimens, despite reporting a decline in their tensile properties.

Some works combine vapor smoothing with other finishing techniques. For instance, Nguyen et al. [23] carried out a design-of-experiments-based investigation on the treatment of ABS parts combining an acetone-based chemical treatment, drying, and aluminum coating, observing a decrease in surface roughness and heat absorption of radiative heating. Maciag et al. [24] performed a study on the influence of acetone smoothing and subsequent galvanic copper plating over the surface parameters of ABS prints. Studies considering the feasibility of laser polishing for FFF PLA parts include the ones presented by Chen et al. [25] and Moradi et al. [26].

Regarding thermal treatments, one can find more published data concerning the treatment of semicrystalline polymers such as PLA. For example, an increase in the crystallinity degree through thermal annealing over the glass transition temperatures (T_g_) of PLA samples was reported by Wach et al. [27]. This enhancement favorably impacted the flexural stress of the samples by an average of 14%. Improvements in interlayer tensile strength of the same polymer were reported by Bhandari et al. [28]. Increased inter-laminar toughness of specimens made from amorphous ABS when these were treated over T_g_ was noted by Hart et al. [29]. In these studies, though, less attention is put into evaluating the surface characteristics of the treated parts.

A relevant study concerning the post-processing of polyetherimide (PEI) parts was recently presented by Zhang et al. [30]. They used thermal annealing to post-process the samples and noted a relaxation of thermal stresses when PEI specimens were treated for long periods at temperatures only below its T_g_. 

Mechanical post-processing methods aim to replicate conventional metal finishing techniques applied to thermoplastics by cutting or pressing the peaks of the outer profile of the manufactured parts. The treatment of complex or intricate shapes using these processes may seem challenging, but some recent studies have shown progress in this direction [31,32]. Machining, barrel finishing, ball burnishing, and sanding are examples of mechanical post-processing finishes found in the published literature. In particular, Boschetto et al. [33] conducted an experimental analysis and designed a theoretical model to study the integration between FFF technology and barrel finishing (BF) to improve the surface quality of printed parts. A significant contribution of the study was that BF’s action is deeply affected by the deposition angle of the initial profiles of the substrates. The same research group explored the finishing of FFF parts by computer numerical control [34]. They managed to set the cutting depth as a function of the deposition angle and reported a reduced average roughness and reliable uniformity of finished surfaces. Mali et al. [35] proposed the use of abrasive flow for the finishing of FFF ABS parts. The process was carried out with a self-synthesized abrasive media made of marble powder and Karanja oil and increases in the average surface roughness were encountered with the increase in active cutting particles and extrusion pressure.

One of the few comparative studies regarding different post-processing techniques applied to AM parts was published by Nsengimana et al. [36]. Differences between tumbling, shot peening, hand finishing, spray painting, CNC machining, and chemical treatment on the dimensional accuracy of laser-sintered Nylon and Alumide^®^, as well as FFF ABS parts, were investigated, and the advantages and disadvantages of each of these methods discussed.

As shown, most of the references focus on the post-processing of readily available materials, often used for prototyping, i.e., PLA or ABS. However, there is a scarcity of published research that considers the post-processing of higher performance thermoplastics. Some industries, such as aerospace, biomedical, and automotive, have adopted AM manufacturing technologies beyond prototyping to produce intermediate tooling and end-use parts [37]. These sectors often require products capable of working at elevated temperatures, in the presence of flames or harsh solvents, and under high mechanical loads [38]. In this regard, the engineering-grade thermoplastic such as ULTEM™ 9085 (Ultem) offers a remarkable potential opportunity to fulfill the industries’ needs, owing to its unique combination of high mechanical properties [39] and flame, smoke, and toxicity rating [40]. 

Based on the above, this work aims to investigate a series of post-processing techniques to treat FFF Ultem specimens, namely vapor smoothing, chemical solvent immersion, high-temperature thermal annealing, ball burnishing, abrasive shot blasting, and shot peening, providing optimized process parameters and a comparative overview of the applicability and effect regarding the surface quality enhancement.

## 2. Materials and Methods

### 2.1. Manufacturing of the Samples

The engineering-grade thermoplastic ULTEM™ 9085 (Ultem) was chosen as a model material to fabricate all samples in a Fortus^®^ 400mc professional fused filament fabrication (FFF) printer (Stratasys Ltd., Edina, MN, USA). This printer is equipped with a thermally controlled chamber that ensures a stable temperature of 195 °C during the printing process. Rectangular solid parts (infill of 100%) were printed with a flat surface of 10 × 127 mm^2^, a height of 4 mm, a 0.254 mm layer height, ±45° raster angle, one external contour, and a flat horizontal orientation. Three repetitions were fabricated for each studied post-treatment.

### 2.2. Chemical Post-Processing

#### 2.2.1. Vapor Smoothing

The first treatment was intended to improve the surface quality of Ultem parts through the partial dissolution of their outer layer due to prolonged contact with a chemical vapor (a process known as vapor smoothing). Due to the low compatibility between Ultem and chlorinated solvents [41], and its low boiling point (around 61 °C), chloroform (purity of 99.9%) was chosen for the smoothing process. 

Vapor smoothing was performed by placing the substrates on an elevated sample bed (whose function was to avoid direct contact between the liquid solvent and the parts) within a 200 × 130 × 60 mm glass container. Next, 50 mL of chloroform were added to the bottom of the container, which was then sealed for 120 min. After the 120-min cycle, samples were removed and allowed to dry in ambient laboratory conditions for at least 24 h before further processing. Finally, two more sets of samples were treated for 180 and 270 min, respectively.

#### 2.2.2. Support Removal Solvent 

The second chemical treatment was aimed at analyzing the surface integrity of Ultem parts after being treated with a solvent mixture capable of dissolving polysulfone, a thermoplastic commonly used as support material for Ultem in FFF. The effect of this treatment on the mechanical performance of Ultem and the optimal treatment time has been previously demonstrated [42].

Samples were submerged in a mixture of equal volumetric parts of 1,4-dioxane and toluene for a period of 4 hours. A similar setup as the one used for the vapor smoothing was used. This time, though, samples were fully immersed in the solvent (the volume used was 400 mL), and the liquid mixture was constantly agitated using a magnetic stirrer. After the treatment cycle, samples were removed and allowed to dry in a vacuum chamber for at least 12 h before further processing.

### 2.3. Thermal Annealing

A differential scanning calorimetry (DSC) of Ultem enabled identifying its glass transition temperature at 181 °C. This analysis was performed using a DSC821 measuring device from Mettler Toledo and a heating rate of 10 K·min^−1^. The lack of other phase transitions such as crystallization or melting, indicated the amorphous nature of the studied polymer. A sudden release of energy at 370 °C revealed a loss of integrity and irreversible degradation of the material. Another point to consider is that, during manufacturing, the material is exposed to a temperature of 195 °C for long periods inside the printing chamber. For these reasons, temperatures of 210 °C, 225 °C, and 240 °C were chosen as annealing temperatures. Thermal annealing of Ultem samples was performed at the set temperature for 30 min in an electric furnace from Hobersal, preheated before introducing the samples. Once the treatment was completed, cooling was performed at a constant rate of 1.5 °C·min^−1^ until it reached a temperature of 150 °C to minimize thermal stresses. Then, the cooling rate was increased to 8 °C·min^−1^ until room temperature.

### 2.4. Mechanical Post-Processing

#### 2.4.1. Ball Burnishing

Ball burnishing is a finishing operation based on the plastic deformation of a workpiece’s surface via the application of a hard, highly polished ball subjected to a constant external force. Ball burnishing of Ultem specimens was conducted with a burnishing tool designed to be coupled to a CNC milling machine. This tool has an innerspring, the stiffness of which determines the range of ball burnishing forces exerted by a 10 mm chrome-hardened steel ball. The process was made following the procedure described in a previous publication [43] using the optimal configuration of a force of 400 N, 10 passes of the tool, a lateral path width of 0.32 mm, and a forward speed of 2000 mm·min^−1^. Samples were ball burnished on the top side.

#### 2.4.2. Abrasive Shot Blasting

Shot blasting is a cold surface treatment that involves projecting beads on the workpiece to change its surface state, causing plastic deformation to prepare the surface for subsequent processes such as applying a coating layer. In the present study, shot blasting was performed in a manually operated pressure-controlled sandblasting cabinet. The working pressure was set at 5 bar, and samples were individually blasted for 10 to 20 s, using either a mix of spherical glass beads (100 to 850 µm) or white corundum (220 to 36 FEPA (Federation of European Producers of Abrasives) grit, or about 53 to 500 µm). The angle of incidence was kept between 75° and 90°.

#### 2.4.3. Shot Peening

The process of shot peening resembles abrasive shot blasting but uses metallic beads to treat the surface of the samples. In the present case, a small hole was drilled at one end of the samples to facilitate their hanging on a rotary unit and allowing the shot peening process in an automated chamber. Spherical stainless-steel beads with diameters ranging from 0.2 to 0.3 mm were propelled at the samples using a 2500 rpm turbine working at a pressure of 5 bar during a total treatment time of 10 min. 

A summary of the post-processing techniques and experimental conditions used in the present study can be found in Table 1.

### 2.5. Dimensional Accuracy and Surface Roughness

The effects of each post-processing technique on the surface of the FFF parts were firstly investigated through changes in their width, height, and length dimensions. Secondly, changes in mass and surface roughness were also examined. The latter was evaluated in terms of the average roughness (*R_a_*) and the average maximum peak-to-valley height of the roughness profile (*R_z_*), using a portable roughness gauge Rugosurf 20 from Tesa Technology. The sampling length and cut-off values were set based on the ISO 4288:1996 standard [44].

The surface state of the treated samples was captured using an Olympus DSX1000 digital microscope (Olympus Iberia, Barcelona, Spain) fitted with a DSX10-SXLOB lens and using a 1× and 20× magnification. Surface morphology was examined with a JSM-6460 Scanning Electron Microscope (SEM)(JEOL Ltd., Akishima, Japan). 

## 3. Results and Discussion

### 3.1. Mass and Dimensional Changes

The mass and dimensional changes of all specimens subjected to the different post-processing techniques are summarized in Table 2. Differences in mass, height, width, and length are given as absolute values.

Overall, solvent support removal, abrasive shot blasting, and shot peening have not induced substantial changes in the mass or the main dimensions of the treated parts. This means that there is no detectable solvent absorption or Ultem dissolution during the support removal process. In addition, abrasive mechanical post-processes, performed under the previously described treatment conditions, do not abrase the material enough to induce detrimental changes in their shape.

Regarding vapor smoothing, it is the only treatment that has induced a significant weight increase in the samples. This change is more pronounced with increased treatment times, indicating a gradual absorption of chloroform vapors. Considering the initial mass of the samples was around 6 grams, the 0.35-gram increase reported after 270 min of treatment represents a change of almost 6%. A point to note concerning the dimensional analysis of this particular treatment is that, during the first 120 min, there is a slight reduction in all dimensions due to an initial smoothing of the outer surface as adjacent Ultem filaments fuse. As time progresses, despite retaining their overall shape, samples expand or swell in all directions. With even more prolonged exposure, samples would probably start to completely melt and lose their shape, which would be highly undesirable.

In terms of dimensional accuracy, specimens subjected to thermal annealing suffered the most remarkable transformation: they all expanded in the building direction (an average of 0.5 mm in height) and contracted in the remaining directions (1 and 5 mm in width and length, respectively). This correlates with the theory proposed by Zhang et al. [30] concerning the relaxation of thermal stresses created in the building direction, which seems to be valid at temperatures higher than the material’s T_g_ as long as exposure is maintained within the herein presented ranges. It is also noteworthy to mention that higher temperatures induce more significant dimensional changes.

Finally, ball burnished samples experienced a decrease in height (coinciding with the ball burnishing direction) and a moderate expansion in width and length. As demonstrated in previous publications [43], part of the applied burnishing force is used to allocate the material in the existing inner voids, resulting in surface densification. Therefore, the increase in width and length should not be attributed to an overall deformation of the part but to creating an overhanging edge due to the expansion of the pressed material on the upper layer.

### 3.2. Surface Roughness Evaluation

As discussed in the introduction, the surface roughness of an additively manufactured part tends to play a crucial role in determining its overall performance. A smoother surface will usually imply an enhanced mechanical performance (superficial defects tend to act as crack nucleation sites) and will also be better received by the end consumer as parts resemble their conventionally manufactured counterparts. 

**Figure 1:**
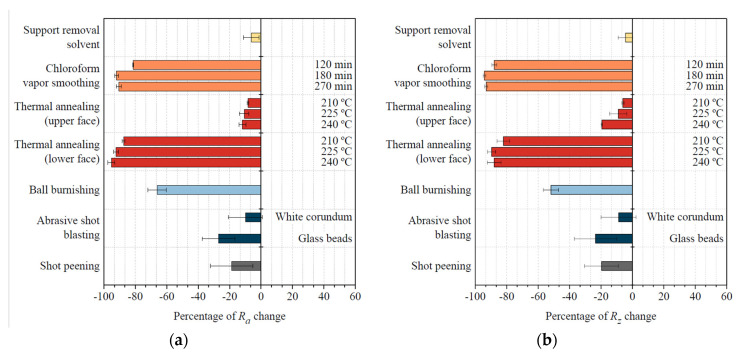
(**a**) Changes in average surface roughness; (**b**) Changes in average peak-to-valley height of the roughness profile.

In this sense, the analysis of the surface roughness parameters *R_a_* and *R_z_* presented in Figure 1 shows that all finishing techniques herein presented have lowered the surface roughness of FFF Ultem parts to a certain degree. However, an exception should be made in the chemical treatment to remove Ultem’s support material, as changes in surface roughness are almost negligible. This result was expected as the purpose of this treatment is to affect Ultem’s integrity as little as possible.

Results obtained from the other chemical treatment, namely vapor smoothing with chloroform, are drastically different. Improvements on Ultem’s surface roughness are as remarkable as 90–95% (*R_a_* = 1.4 µm, *R_z_* = 3.57 µm) after 180 min of treatment. Interestingly, there is no further improvement after this point (surface roughness after 270 min is similar to the one obtained after 180 min), while chloroform continues to be absorbed, as discussed in the mass and dimensional analysis section. For this reason, 180 min should be considered the maximum treatment time to achieve an excellent surface finish. The magnitude of the roughness improvements reported by Chohan et al. [17] (99.62%) is comparable to the results of the present study. It should be noted, though, that the initial surface roughness of their studied hip replica was two-times lower than the one of the Ultem parts used in this study and that their total contact time with the vaporized solvent was considerably lower (in the order of a 1 min) than in the present case. The roughness improvements reported by Rajan et al. [22] are more moderate (around 80%) when they used tetrahydrofuran (THF) vapors during a total time of 5 min to smooth FFF PLA parts. They also observed that the initial build orientation (and thus the initial surface roughness) plays a crucial role in the outcome of the vapor smoothing process. Considering that all consulted treatment times are lower than the ones needed to obtain similar results with Ultem, it is safe to affirm that Ultem’s chemical resistance to chloroform is higher than the chemical resistance of ABS to acetone and of PLA to THF.

Concerning thermal annealing, no significant differences in surface roughness have been identified, regardless of the temperature of the treatment. Nevertheless, dimensional changes are higher at higher temperatures, implying that there is no benefit in increasing the temperature of the treatment from 210 °C to 240 °C. The obtained 15% improvement in the surface roughness of the upper part (*R_a_* = 12.60 µm, *R_z_* = 62.28 µm) is probably due to the partial fusion of adjacent filaments. Interestingly, the lower part of the samples, which was in direct contact with the thermal chamber’s tray, presented a glass-like finish, with improvements in *R_a_* higher than 90% (1.05 µm) and higher than 80% in and *R_z_* (8.69 µm). This suggests that a physical contact that favors heat transfer by conduction is noticeably more effective in improving the surface finish of the parts than heat transfer by convection, as it happens on the air-exposed faces of the part.

Surface roughness obtained after mechanical post-processing of the parts differs considerably. When samples are ball burnished, *R_a_* and *R_z_* diminish by about 70% (5.06 µm) and 50% (34.19 µm), respectively, due to the plasticization of the outer layer because of the forced pressing of the peaks by the burnishing ball. This technique uses a stainless-steel tool that can be adapted to a numeric control machine, making it an easily automatable process, but presents some challenges when the totality of the part needs to be treated, or when the part has difficult-to-access zones. With abrasive shot peening or abrasive shot blasting using glass beads, the surface roughness has improved by 20% to 25% (*R_a_* = 10.83 µm, *R_z_* = 3.57 µm). The use of white corundum as blasting media has resulted in a more moderate improvement of around 10% (*R_a_* = 13.40 µm, *R_z_* = 62.54 µm). A noticeable fact is that the standard deviation of the roughness measurements after these finishing processes were applied to Ultem parts is considerably higher than with the other treatments, which indicates more limited repeatability as they depend on the experience of the operator performing the treatment, the contact time, and the wear of the beads, amongst others. In comparison with the 50% improvement in surface roughness reported by Valerga Puerta et al. [45] when they corundum blasted FFF PLA parts using analogous treatment time conditions as the ones reported in the present study, Ultem appears to have an increased initial toughness that could explain such more moderate improvements. 

### 3.3. Surface Analysis

**Figure 2:**
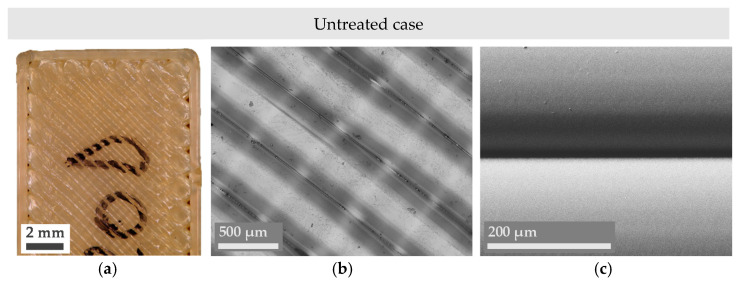
Digital microscope (**a**,**b**) and SEM micrographs (**c**) of untreated Ultem parts.

**Figure 3:**
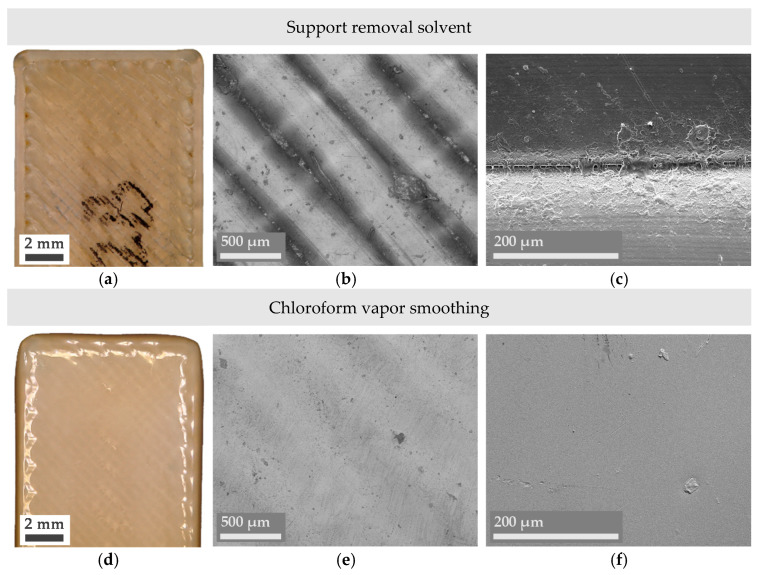
Digital microscope (**a**,**b**,**d**,**e**) and SEM micrographs (**c**,**f**) of the chemically treated Ultem parts.

**Figure 4:**
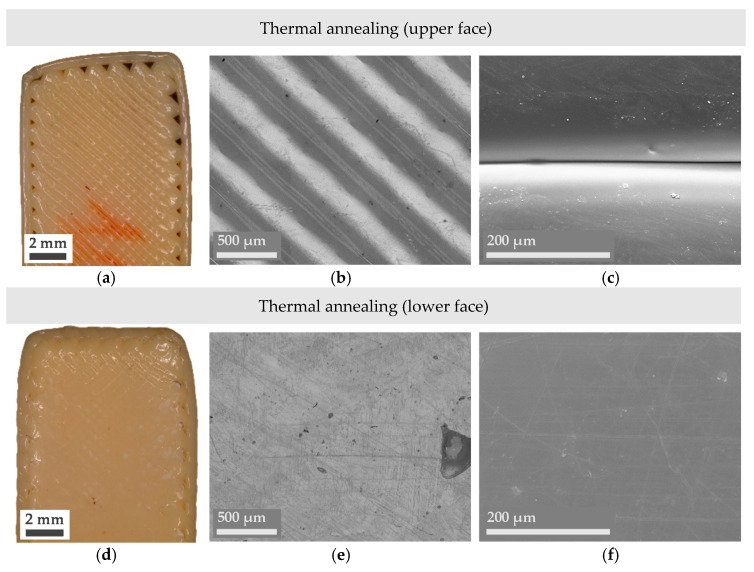
Digital microscope (**a**,**b**,**d**,**e**) and SEM micrographs (**c**,**f**) of the thermally treated Ultem parts.

**Figure 5:**
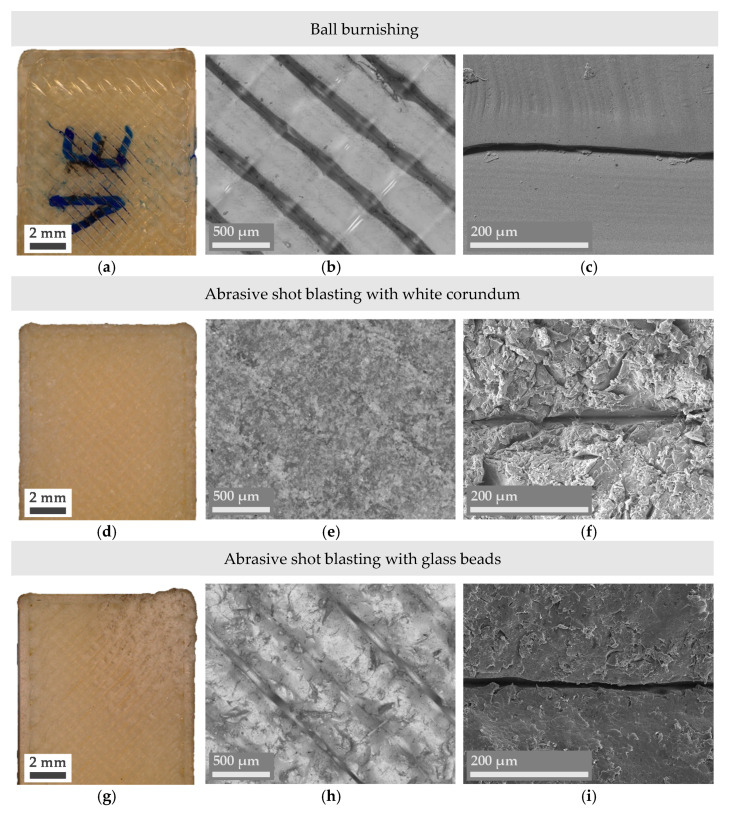

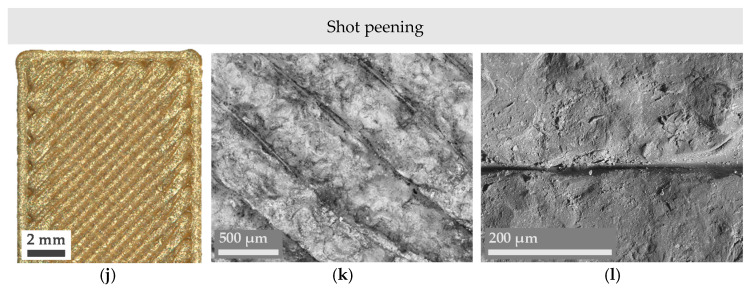
Digital microscope (**a**,**b**,**d**,**e**,**g**,**h**,**j**,**k**) and SEM micrographs (**c**,**f**,**i**,**l**) of the mechanically treated Ultem parts.

Even though obtaining a combination of lower *R_a_* and *R_z_* values is typically enough to affirm an improvement in the surface finish of a part, microscope imaging provides a deeper insight into the reasons behind surface changes and their nature. In this sense, Figures 3–5 present a series of optical microscope images and SEM micrographs aimed at the direct comparison between the macroscopic and microscopic state of the treated samples. Images of an untreated or pristine part have also been added in Figure 2.

Figure 3a reveals that, compared to the pristine case, Ultem has lost some of its characteristic shine after being submerged in a mixture of 1,4-dioxane and toluene for 4 h. This tonality change could be explained by the presence of an Ultem residue that has been partially dissolved and non-uniformly redeposited along the interfilament space (some zones are more coalesced, but some are not), as the more magnified central and right images (Figure 3b,c) show.

As the roughness analysis has revealed, a drastically different surface is obtained when Ultem is vapor-smoothed in the presence of chloroform (Figure 3d–f). Microscopy results display a smooth surface where the additively manufactured nature of the part is almost inappreciable as Ultem filaments from the outer surface have completely melted and resolidified, forming a uniform layer. This result confers perspective and validates the effectiveness of the other analyzed chemical treatment: In comparison with vapor smoothing, Ultem’s surface is much less affected despite being in direct contact with a chemical mixture capable of dissolving a support material with a similar chemical structure to Ultem’s.

Regarding the visual inspection of the thermally annealed samples in Figure 4, the overall shape of the specimen is lightly modified as the corners appear more rounded, and the upper edge of the part has lost its perpendicularity with the other edges. Nonetheless, magnified images (Figure 4b,c,e,f) demonstrate that the air gap between adjacent filaments has diminished in the upper face and has almost completely disappeared in the lower face. Taking this into consideration, further experiments should be conducted at a slightly lower temperature (lower than 210 °C, but higher than the material’s T_g_) to try to reduce dimensional changes while still achieving better filament adhesion. Ideally, the presence of a solid or liquid media that enters in direct contact with the totality of the part during the thermal treatment would help achieve an even smoother surface, as detected in the inspection of the lower side of the thermally annealed part.

Figure 5a–c demonstrates how the characteristic rounded shape of FFF manufactured parts is modified due to the ball burnishing process, as predicted from the roughness analysis. Ultem filaments appear flattered, and the overall state of the filament’s surface, not damaged. On the contrary, the surface of the mechanically treated parts using abrasive treatments shown in Figure 5d–l appears to be radically modified. While the average surface roughness or overall dimensions of the treated parts do not reveal such intense changes, the surface of a single filament has been deeply affected.

Samples that have been shot blasted with white corundum (Figure 5d–f) have suffered the most remarkable changes. The impact of small, non-rounded beads made of a material as hard as corundum has eroded the surface and completely changed its morphology. The Ultem part’s external filaments show cracks and numerous irregularities; Ultem has lost its shine, and the part appears more mattified. Nevertheless, the presence of filaments looks more blurred, meaning that the overall aspect of the part is more uniform. This can be regarded as a positive outcome, as a specific surface morphology can be desirable to promote adhesion of further coatings that want to be applied to the part.

In the case of abrasive shot blasting with glass beads (Figure 5 g–i), the surface is not as eroded as with white corundum due to the softer nature of the used abrasive and its rounded shape, but the surface still shows noticeable alterations in the form of small protrusions generated by the beads’ impact. It should be remembered that initially spherical glass beads tend to break down into smaller and more protruded parts during the shot blasting process. Something to mention is that the right corner of the treated part shown in the macroscopic image Figure 5g has been more damaged than the rest of the surface, revealing one of the main drawbacks of these abrasive techniques: the need to automate or very precisely control the exact time, incidence angle, and distance of the abrasive gun. While in the case of chemical or thermal treatments a slight time deviation in the duration of the treatment is perfectly acceptable, in the case of abrasive shot blasting a few seconds’ deviation can be detrimental to the final result.

Finally, microscopy images from the shot peened samples (Figure 5j–l) show a shinier surface due to the presence of metallic residue from the stainless-steel beads. Interestingly, despite the longer duration of this treatment (10 min versus 10 to 20 s in the case of abrasive shot blasting), it has resulted in a more uniform surface due to its more controlled nature (shot peening was performed in an automated chamber and with a higher distance between the gun and the samples). In addition, the impact of spherical metallic beads has induced pressing of the part’s external filaments, which denotes the application of compressive stresses that could be beneficial for the fatigue life of the parts. Considering the simplicity of this process and the insignificant affectation of the parts’ dimensions, shot peening’s effect on the mechanical properties of FFF Ultem parts is a key point that should be addressed in future works.

## 4. Conclusions

In this work, six different post-processing techniques for fused filament fabricated Ultem parts have been proposed, and their effect on dimensional accuracy, mass, and surface roughness of the treated parts, compared. Microscope imaging has provided insight into the reasons behind the observed changes in surface roughness. From the obtained results, the following conclusions can be extracted:

- Overall, chemical and thermal treatments, as well as ball burnishing, have been postulated as valid candidates to significantly enhance the finish of FFF Ultem parts, despite requiring the use of solvents or inducing controlled dimensional changes.
- In particular, chemical vapor smoothing has resulted in the highest improvement in surface roughness, but the absorption of chemical vapors should be taken into consideration.
- Thermally annealed samples above Ultem’s glass transition temperature retain their overall shape but have expanded in the building direction (indicating thermal stresses release) and contracted in width and length.
- Considering the observed differences between the upper and the lower side of the thermally treated samples, the addition of a physical media to conduct heat to the samples (instead of air convection) is expected to be beneficial to obtain a more uniform surface roughness. As dimensional changes increase with the treatment temperature, the use of a slightly lower temperature than the minimum 210 °C used in this study should also be considered. Ball burnishing has resulted in the best equilibrium between an improved surface roughness and minimal dimensional changes.
- The effects of abrasive shot blasting and abrasive shot peening are more moderate at a macroscopic scale but have modified the parts’ surface morphology to the greatest extent.

Future works should focus on studying the affectation of the proposed finishing techniques on Ultem’s mechanical performance and searching mathematical coefficients to predict dimensional and roughness changes.

## Figures and Tables

**Table 1:** Post-processing techniques and procedures applied to Ultem pieces.

Post-Processing Technique	Fixed Experimental Conditions	Variable Experimental Conditions
Vapor smoothing	Solvent: Chloroform vapors Temperature: ambient laboratory conditions	Exposure times: 2, 3 and, 4.5 h
Support removal solvent	Solvent: equivolumetric mixture of 1,4-dioxane and toluene Temperature: ambient laboratory conditions	
	Constant agitation using a magnetic stirrer Exposure time: 4 h	
Thermal annealing	Preheating of the furnace prior to the introduction of the samples Exposure time at the set temperature: 30 min Cooling rate: 1.5 °C·min^−1^ until 150 °C, 8 °C·min^−1^ until room temperature	Temperatures: 210, 225, and 240 °C
Ball burnishing	Ball burnishing force: 400 N Number of passes of the tool: 10 Lateral path width: 0.32 mm Forward speed: 2000 mm·min^−1^	
Abrasive shot blasting	Pressure: 5 bar Treatment time: 10–20 s	Abrasive media: White corundum, and glass beads
Shot peening	Spherical stainless-steel beads Pressure: 5 bar Treatment time: 10 min	

**Table 2:** Mass and dimensional changes as a function of the post-processing treatment.

Post-Process	Details	Δ Mass [g]	Δ Height [mm]	Δ Width [mm]	Δ Length [mm]
Support removal solvent	-	+0.03 ± 0.05	−0.04 ± 0.05	−0.01 ± 0.01	−0.02 ± 0.03
Chloroform vapor smoothing	120 min	+0.12 ± 0.01	−0.05 ± 0.01	−0.10 ± 0.04	−0.18 ± 0.02
	180 min	+0.22 ± 0.01	+0.01 ± 0.05	+0.05 ± 0.02	−0.14 ± 0.02
	270 min	+0.35 ± 0.03	+0.10 ± 0.02	+0.15 ± 0.03	+0.16 ± 0.01
Thermal annealing	210 °C	+0.00 ± 0.01	+0.48 ± 0.12	−0.88 ± 0.19	−4.73 ± 0.84
	225 °C	−0.02 ± 0.01	+0.52 ± 0.18	−0.93 ± 0.26	−5.11 ± 1.38
	240 °C	−0.01 ± 0.00	+0.62 ± 0.04	−1.07 ± 0.06	−5.73 ± 0.49
Ball burnishing	-	+0.00 ± 0.03	−0.11 ± 0.02	+0.08 ± 0.03	+0.30 ± 0.09
Abrasive shot blasting	White corundum	+0.01 ± 0.02	−0.01 ± 0.06	+0.02 ± 0.03	+0.08 ± 0.07
	Glass beads	−0.01 ± 0.01	−0.05 ± 0.02	+0.02 ± 0.02	+0.03 ± 0.07
Shot peening	-	−0.02 ± 0.01	−0.02 ± 0.01	+0.01 ± 0.02	+0.05 ± 0.06
